# The interplay of psychosis and victimisation across the life course: a prospective study in the general population

**DOI:** 10.1007/s00127-017-1430-9

**Published:** 2017-08-31

**Authors:** Steven Honings, Marjan Drukker, Margreet ten Have, Ron de Graaf, Saskia van Dorsselaer, Jim van Os

**Affiliations:** 1 Department of Psychiatry and Psychology, South Limburg Mental Health Research and Teaching Network, Maastricht University Medical Centre, Maastricht, The Netherlands; 2 Netherlands Institute of Mental Health and Addiction, Utrecht, The Netherlands; 3 King’s Health Partners, Department of Psychosis Studies, Institute of Psychiatry, King’s College London, London, UK; 4 Department of Psychiatry, Brain Center Rudolf Magnus Institute, University Medical Center Utrecht, PO BOX 85500, 3508 GA Utrecht, The Netherlands

**Keywords:** Psychosis, Psychotic experience, Violence, Childhood trauma, Victimisation

## Abstract

**Purpose:**

Psychosis has been associated with adult victimisation. However, it remains unclear whether psychosis predicts incident adult victimisation, or whether adult victimisation predicts incident psychosis. Furthermore, a moderating effect of childhood victimisation on the association between psychosis and adult victimisation has not been investigated.

**Methods:**

The longitudinal association between baseline psychotic experiences and six-year incidence of adult victimisation was assessed in a prospective general population cohort of 6646 adults using logistic regression analysis. The association between baseline adult victimisation and six-year incidence of psychotic experiences was examined as well. Furthermore, the moderating effect of childhood victimisation on these bidirectional associations was analysed.

**Results:**

Psychotic experiences and childhood victimisation were both associated with an increased risk of incident adult victimisation. However, this was through competing pathways, as suggested by a negative interaction between psychotic experiences and childhood victimisation. Baseline adult victimisation and childhood victimisation both independently increased the risk of incident psychotic experiences, but there was no interaction between adult victimisation and childhood victimisation.

**Conclusions:**

Psychosis and victimisation are interconnected throughout the life course. Childhood victimisation is connected to psychosis through two pathways: one direct and one indirect through adult victimisation. In individuals without childhood victimisation, psychosis and adult victimisation bidirectionally impact on each other.

## Introduction

Psychosis has been associated with an increased risk of violence perpetration [1–5]. However, contrary to the common stereotype that individuals with severe mental illness are dangerous [6], evidence shows that these individuals are more likely to be victims of violence than perpetrators of violence [7, 8]. Among individuals with psychosis, victimisation is prevalent [9], both during childhood [10–12] and adulthood [13–15].

Various forms of childhood victimisation, including sexual abuse, physical abuse, emotional abuse [16] and being bullied [17], have been associated with psychosis in the literature [10–12]. Childhood victimisation has been associated with both psychotic experiences (PE) [10] and full-blown psychotic disorder [11, 12], thus covering the complete spectrum of the extended psychosis phenotype [18–20]. Most research to date has focussed on the hypothesis that childhood victimisation is a risk factor for the development of psychosis [16]. However, evidence shows that PE increase the risk of incident childhood victimisation as well, thereby showing that the association between childhood victimisation and PE is bidirectional [10].

Recently, studies found evidence that psychosis is associated with adult victimisation as well. Compared with general population individuals, the prevalence of criminal and violent victimisation among individuals with psychosis and other severe mental illnesses was high [13–15]. However, the nature of this association remains unclear, since most studies to date had some methodological limitations. First, most studies to date used cross-sectional study designs to examine the association between psychosis and adult victimisation [13–15]. Therefore, it is unclear whether psychosis increases the risk of incident adult victimisation or vice versa. To our knowledge, only one longitudinal study examined the association between adult victimisation and psychosis, showing that adult adversities were associated with an increased risk of incident PE [21]. However, no longitudinal study to date examined whether psychosis predicts incident adult victimisation. Thus, it remains unknown whether the association between psychosis and adult victimisation is bidirectional, similar to the association between psychosis and childhood victimisation [10]. Second, few studies examined the influence of childhood victimisation on the association between psychosis and adult victimisation [22], while childhood victimisation is associated with an increased risk of both psychosis [10–12] and adult victimisation [23–29]. Previous studies have shown that childhood victimisation and various environmental factors combine synergistically to increase the risk of PE over and above their isolated products [21, 30–32]. However, no previous study examined whether psychosis predicts incident adult victimisation, while simultaneously examining the potential moderating effect of childhood victimisation.

**Fig. 1:**
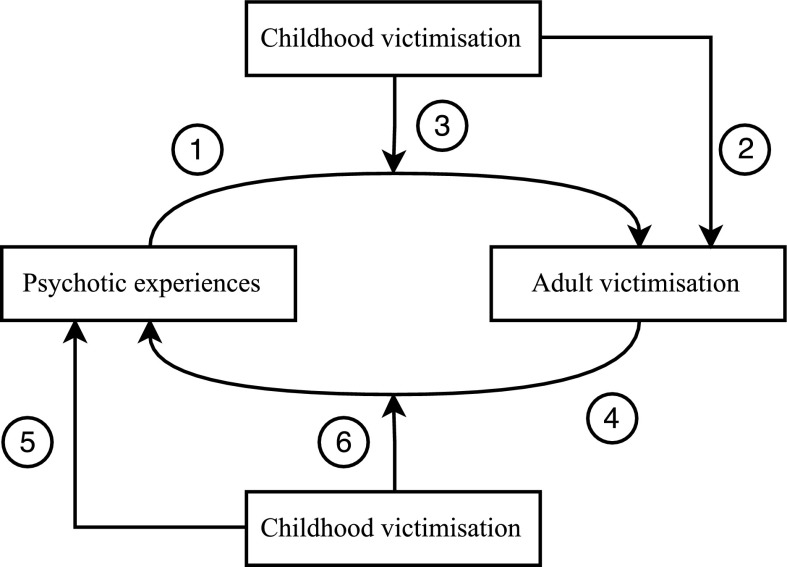
Hypotheses relating to the bidirectional association between psychotic experiences and victimisation

The present study aims to bridge these gaps in the literature. In line with the research on childhood victimisation and PE, we hypothesized that there would be a bidirectional association between PE and adult victimisation, that is moderated by the presence of childhood victimisation (Fig. 1). More specifically, we hypothesized that: (1) PE are associated with incident adult victimisation; (2) childhood victimisation is associated with incident adult victimisation; (3) the co-occurrence of PE and childhood victimisation predicts a stronger association with incident adult victimisation than the product of their isolated effects; (4) adult victimisation is associated with incident PE; (5) childhood victimisation is associated with incident PE; (6) the co-occurrence of adult victimisation and childhood victimisation predicts a stronger association with incident PE than the product of their isolated effects.

## Methods

### Sample

This study uses data pertaining to the second Netherlands Mental Health Survey and Incidence Study (NEMESIS-2), a longitudinal study of the prevalence, incidence, course and consequences of mental disorders in the Dutch general population [33]. Participants were selected based on a multistage random sampling procedure. At baseline (T0), 6646 persons aged 18–64 years were interviewed with the Composite International Diagnostic Interview (CIDI) version 3.0, a fully structured lay-administered diagnostic interview generating DSM-IV diagnoses [34]. At follow-up, respectively, three (T1, *N* = 5503) and 6 years (T2, *N* = 4618) after baseline, subjects were re-interviewed. A more comprehensive description of the design can be found elsewhere [33]. In the present analyses, only individuals who responded to all three assessments were included.

### Psychotic experiences

PE were assessed at baseline (T0) and both follow-up measurements (T1, T2) using a psychosis add-on instrument based on the sections of psychotic symptoms in CIDI versions 1.0 and 2.0. The instrument consisted of 20 questions regarding lifetime PE. The 20 items included 15 delusional experiences and 5 hallucinatory experiences, described in detail elsewhere [35]. Individuals with at least one lifetime PE were contacted for a re-interview by telephone. Re-interviews were conducted by an experienced clinician at the level of psychologist or psychiatrist, within 8 weeks after the initial interview using questions from the Structured Clinical Interview for DSM-IV. Findings from all re-interviews were discussed with a second clinician [35]. PE were defined as clinically validated when the psychotic nature of the self-reported PE was confirmed at clinical re-interview. The response rates for the re-interviews at the three time points were 73% (T0), 84% (T1) and 81% (T2), respectively. At baseline, lifetime PE were assessed. At both follow-up surveys, participants were asked about PE since the last interview. For the present analyses, only clinically validated PE were used. Individuals with self-reported PE who could not be reached for re-interview were excluded from the analyses. PE were defined present if the participant had at least one clinically validated PE. Incident PE was defined present if a participant reported at least one clinically validated PE at one of the follow-up surveys, but reported no PE at baseline.

### Childhood victimisation

At baseline, childhood victimisation was assessed retrospectively. Participants were asked whether they had experienced emotional abuse, psychological abuse, physical abuse or sexual abuse before the age of 16 years. Consistent with previous analyses [7], childhood abuse was defined present if a participant had experienced psychological abuse or emotional abuse on two or more occasions, or physical abuse/sexual abuse on one or more occasion. In addition, being bullied was assessed at baseline by asking participants if they had been bullied regularly before the age of 16 years. For the present study, childhood victimisation was defined present if a participant reported childhood abuse or bullying at baseline. Individuals with missing data on childhood victimisation were excluded from the present analyses (*n* = 140).

### Adult victimisation

At baseline, participants were asked about lifetime violent and psychological victimisation by an intimate partner. In addition, lifetime sexual victimisation by any person in general since the age of 16 years was assessed. To increase the likelihood of these forms of victimisation being reported, the interviewer did not mention any type of victimisation during the interview. Instead, different forms of victimisation were listed and numbered in a booklet. Participants were asked to provide the numbers of the type of victimisation. Psychological victimisation included name-calling, offending, belittling, punishing unjustly, blackmailing and threatening. Physical victimisation included kicking, biting, hitting, trying to wound with an object (gun, knife, piece of wood, pair of scissors, other object) or hot water. Sexual victimisation included unwanted touching, forced undressing and forced sexual activity. Consistent with previous work [7, 36], psychological victimisation was defined present if it occurred on two or more occasions, and violent/sexual victimisation on one or more occasions.

At both follow-up measurements, participants were asked about violent, psychological and sexual victimisation since the last assessment by any person in general, and if so, by whom (i.e. partner, ex-partner, family member, acquaintance, stranger). To reach consistency with the baseline questions, physical victimisation and psychological victimisation at follow-up were defined present if the respective form of victimisation was perpetrated by an intimate partner. Sexual victimisation at follow-up was defined present if it was perpetrated by any person in general, conform baseline measurement. The frequencies for all victimisation outcomes were similar to the frequencies at baseline.

Incident physical victimisation by a partner (hereafter: physical victimisation), incident psychological victimisation by a partner (hereafter: psychological victimisation) and incident sexual victimisation (hereafter: sexual victimisation) were defined as present if the participant reported the respective type of victimisation at any of the follow-up interviews, while participants with the respective type of victimisation at baseline were excluded. In addition, a summary variable (any incident adult victimisation) was generated to identify participants who experienced any form of adult victimisation at follow-up, but had no victimisation at baseline. Participants without a partner at baseline and any of the follow-up measurements were excluded when analysing physical victimisation, psychological victimisation or the summary variable.

### Confounders

Age, gender, low socio-economic status, past criminal activity and substance use disorders were hypothesized to be confounders in the present analyses [13, 37]. Arrest was used as a measure of criminal activity. Self-reported arrest was obtained at baseline by asking participants if they had ever been arrested. In addition, CIDI 3.0 was used to define baseline, lifetime diagnoses of any substance use disorder. Finally, household income was a proxy for socio-economic status. The variable included three strata, based on monthly income: low (<€1500), middle (€1500–€3300) and high (>€3300).

### Statistical analyses

All statistical analyses were performed using Stata version 13 [38]. Baseline characteristics were assessed for the complete sample. In addition, subjects with clinically validated PE at baseline were compared with subjects without clinically validated PE, using Chi-square tests and independent sample *t* tests.

Logistic regression analyses were performed to answer the six research questions. All regression models included age, gender, household income (dummies of strata), baseline substance use disorders and arrest as covariates. The numbers of the analyses correspond with the numbers in Fig. 1.

1. Logistic regression analyses were performed to examine the association between baseline PE and incident adult victimisation at follow-up in the complete sample.
2. The association between baseline childhood victimisation and incident adult victimisation was assessed in a separate logistic regression analysis.
3. To assess whether childhood victimisation moderated the association between PE and adult victimisation, a logistic regression analysis was performed using incident adult victimisation as the dependent variable and PE, childhood victimisation and the interaction term PE * childhood victimisation as the independent variables. To any interaction effect, a logistic regression analysis was performed using incident adult victimisation as the dependent variable and a categorical variable containing the following categories as the independent variable, modelled as dummies: (1) No PE, no childhood victimisation (reference group); (2) No PE, childhood victimisation present; (3) PE present, no childhood victimisation; (4) PE present, childhood victimisation present. If the interaction term was below alpha (*α* = 0.10), the associations between PE, childhood victimisation and adult victimisation were analysed stratified by presence or absence of PE and childhood victimisation, respectively.
4. The association between baseline adult victimisation and incident PE at follow-up was examined in a logistic regression analysis in the complete sample.
5. The association between childhood victimisation and incident PE was assessed in a logistic regression analysis in the complete sample.
6. The hypothesized moderating effect of childhood victimisation on the association between baseline adult victimisation and incident PE was examined by conducting a logistic regression analysis using incident PE as the dependent variable and adult victimisation, childhood victimisation and the interaction term adult victimisation * childhood victimisation as the independent variables. A logistic regression analysis was performed using incident PE as the dependent variable and a categorical variable containing the following categories as the independent variable: (1) No childhood victimisation, no adult victimisation (reference category); (2) childhood victimisation present, no adult victimisation; (3) no childhood victimisation, adult victimisation present; (4) childhood victimisation present, adult victimisation present. Again, if the *p* value of the interaction term was below alpha (α = 0.10), the associations between childhood victimisation, adult victimisation and PE were examined in stratified analyses.

## Results

### Baseline characteristics

At baseline, the complete sample included 6359 participants, after exclusion of individuals with self-reported PE who could not be reached for re-interview (*n* = 287). Of these participants, 5.3% (*n* = 340) reported clinically validated PE. More women than men reported PE (Table 1). Moreover, the proportion of individuals with PE differed significantly between the strata of household income. Subjects with PE were overrepresented in the low income group and underrepresented in the high-income group. Furthermore, the baseline prevalence of childhood victimisation, adult victimisation, lifetime substance use disorders and arrest was significantly higher in individuals with PE (Table 1).

**Table 1:** Baseline characteristics of individuals with and without clinically validated PE

	Complete sample	Subjects with PE^a^	Subjects without PE^a^	*t*	*χ* ^2^	*df*	*p* ^b^
Demographics							
*N*	6359	340	6019	–	–	–	–
Number of males (%)	2852 (44.9)	127 (37.4)	2725 (45.3)	–	8.162	1	0.004
Age (SD)	44.4 (12.5)	43.0 (13.2)	44.4 (12.5)	2.049	–	6357	0.041
Household income	–	–	–	–	32.915	2	<0.001
Low	1439 (25.4)	119 (38.4)	1320 (24.7)	–	–	–	–
Middle	2635 (46.5)	133 (42.9)	2502 (46.7)	–	–	–	–
High	1590 (28.1)	58 (18.7)	1532 (28.6)	–	–	–	–
Baseline victimisation							
Childhood victimisation, *N* (%)	2138 (34.4)	207 (61.8)	1931 (32.8)	–	118.161	1	<0.001
Physical victimisation by partner, *N* (%)	614 (10.3)	73 (23.3)	541 (9.6)	–	60.216	1	<0.001
Psychological victimisation by partner, *N* (%)	1716 (28.9)	148 (47.4)	1568 (27.9)	–	55.106	1	<0.001
Sexual violence victimisation, *N* (%)	279 (4.5)	47 (14.1)	232 (4.0)	–	75.956	1	<0.001
Any adult victimisation, *N* (%)	1940 (33.2)	171 (55.0)	1769 (32.0)	–	70.355	1	<0.001
Baseline confounders							
Any lifetime substance use disorder	1037 (16.3)	106 (31.2)	931 (15.5)	–	58.187	1	<0.001
Ever arrested	1346 (21.2)	88 (26.0)	1258 (20.9)	–	4.906	1	0.027

^a^Clinically validated PE

^b^ *p*-value resulting from *t* test or Chi-square test for difference between participants with vs. without PE

### The association between baseline psychotic experiences and adult victimisation

The odds ratio (OR) of the association between baseline PE and any incident adult victimisation was 2.09 (95% CI 0.79–5.56; Table 2). PE were associated with all forms of victimisation, but the OR was only statistically significant for the association between PE and sexual victimisation (OR = 3.51, 95% CI 1.54–7.96).

**Table 2:** Results of logistic regression analyses (ORs and 95% CI) of the association between baseline psychotic experiences, baseline childhood victimisation and incident adult victimisation, adjusted for age, gender, socioeconomic status, self-reported arrest and lifetime substance use disorder

Outcome	Any incident adult victimisation (*n* = 2296)	*p*	Incident sexual victimisation (*n* = 3881)	*p*	Incident physical victimisation by partner (*n* = 2994)	*p*	Incident psychological victimisation by partner (*n* = 2420)	*p*
Separate models								
PE in complete sample	2.09 (0.79–5.56)	0.14	3.51 (1.54–7.96)	<0.01	1.84 (0.42–8.14)	0.42	1.99 (0.75–5.28)	0.17
CV^a^ in complete sample	3.15 (1.87–5.30)	<0.01	2.70 (1.41–5.16)	<0.01	5.49 (2.26–13.34)	<0.01	3.01 (1.74–5.24)	<0.01
Interaction term								
PE * CV in complete sample	0.17 (0.02–1.22)	0.08	N.A.	N.A.	0.12 (0.01–2.32)	0.16	0.16 (0.02–1.12)	0.07
Categorical predictors								
No PE, no CV	Reference	–	Reference	–	Reference	–	Reference	–
No PE, CV present	3.73 (2.16–6.47)	<0.01	2.24 (1.10–4.57)	0.03	7.99 (2.90–22.06)	<0.01	3.47 (1.94–6.21)	<0.01
PE present, no CV	4.49 (1.27–15.90)	0.02	N.A.	N.A.	6.32 (0.71–56.22)	0.10	4.62 (1.30–16.36)	0.02
PE present, CV present	2.88 (0.62–13.39)	0.18	8.72 (3.40–22.32)	<0.01	5.85 (0.64–53.11)	0.12	2.54 (0.55–11.73)	0.23
Stratified models								
PE in subsample without CV	4.81 (1.34–17.29)	0.02	–	–	–	–	4.63 (1.29–16.60)	0.02
PE in subsample with CV	0.73 (0.16–3.37)	0.69	–	–	–	–	0.76 (0.17–3.46)	0.72
CV in subsample without PE	3.76 (2.17–6.53)	<0.01	–	–	–	–	3.49 (1.95–6.26)	<0.01
CV in subsample with PE	0.53 (0.06–4.32)	0.55	–	–	–	–	0.52 (0.07–4.14)	0.54

^a^Childhood victimisation

### The association between baseline childhood victimisation and adult victimisation

Childhood victimisation was associated with all forms of adult victimisation, with ORs ranging from 2.70 (95% CI 1.41–5.16) for sexual victimisation to 5.49 (95% CI 2.26–13.34) for physical victimisation (Table 2).

### The interaction between psychotic experiences and childhood victimisation on the outcome adult victimisation

The interaction term PE * Childhood victimisation was below alpha for the outcome any adult victimisation (Table 2; *p* = 0.08). Follow-up analysis of the interaction between childhood victimisation and PE on the outcome adult victimisation, using a categorical predictor, showed that isolated PE (OR = 4.49, 95% CI 1.27–15.90) and isolated childhood victimisation (OR = 3.73, 95% CI 2.16–6.47) were associated with an increased risk of any adult victimisation. However, the co-occurrence of PE and childhood victimisation was associated with a lower risk of any adult victimisation than their isolated effects (OR = 2.88, 95% CI 0.62–13.39), thus indicating a negative interaction. Analyses in the subsample stratified by presence or absence of childhood victimisation showed that PE were associated with adult victimisation in the subsample without childhood victimisation (OR = 4.81, 95% CI 1.34–17.29). However, in the subsample with childhood victimisation there was no association between PE and adult victimisation. Similarly, childhood victimisation was associated with adult victimisation in the subsample without baseline PE (OR = 3.76, 95% CI 2.17–6.53), but was not associated with adult victimisation in the subsample with baseline PE. Results for the outcomes physical victimisation and psychological victimisation were similar to the results of any adult victimisation and showed a trend towards a negative interaction as well (*p* = 0.16 for physical victimisation, *p* = 0.07 for psychological victimisation). Interaction could not be examined in the model with sexual victimisation as the outcome, because none of the subjects had PE in absence of childhood victimisation. However, the co-occurrence of PE and childhood victimisation (OR = 8.72, 95% CI 3.40–22.32) showed a stronger association with sexual victimisation than isolated childhood victimisation (OR = 2.24, 95% CI 1.10–4.57).

### The association between baseline adult victimisation and incident psychotic experiences

In the complete sample, all forms of baseline adult victimisation were associated with incident PE, with ORs ranging from 1.88 (95% CI 1.34–2.64) for psychological victimisation to 3.77 (95% CI 2.32–6.12) for sexual victimisation, after adjustment for confounders (Table 3).

**Table 3:** Results of logistic regression analyses (ORs and 95% CI) of the association between baseline victimisation, childhood victimisation and incident psychotic experiences, adjusted for age, gender, socioeconomic status, self-reported arrest and lifetime substance use disorder

Outcome	Any incident PE (*n* = 3691)							
Specification of adult victimisation	Any AV	*p*	Sexual victimisation	*p*	Physical victimisation by partner	*p*	Psychological victimisation by partner	*p*
Separate models								
AV^a^ in complete sample	2.28 (1.63–3.20)	<0.01	3.77 (2.32–6.12)	<0.01	2.20 (1.46–3.32)	<0.01	1.88 (1.34–2.64)	<0.01
CV^b^ in complete sample	2.64 (1.90–3.66)	<0.01	2.64 (1.90–3.66)	<0.01	2.64 (1.90–3.66)	<0.01	2.64 (1.90–3.66)	<0.01
Interaction term								
AV * CV in complete sample	0.95 (0.48–1.90)	0.89	0.75 (0.23–2.48)	0.64	0.86 (0.35–2.07)	0.73	1.19 (0.58–2.42)	0.63
Categorical predictor								
No CV, no AV	Reference	–	Reference	–	Reference	–	Reference	–
CV present, no AV	2.38 (1.46–3.89)	<0.01	2.43 (1.71–3.46)	<0.01	2.54 (1.74–3.70)	<0.01	2.30 (1.47–3.59)	<0.01
No CV, AV present	1.94 (1.14–3.31)	0.02	3.64 (1.23–10.74)	0.02	2.09 (1.00–4.36)	0.05	1.41 (0.80–2.49)	0.24
CV present, AV present	4.40 (2.83–6.83	<0.01	6.62 (3.77–11.63)	<0.01	4.54 (2.70–7.64)	<0.01	3.84 (2.49–5.93)	<0.01

^a^Adult victimisation

^b^Childhood victimisation

### The association between baseline childhood victimisation and incident psychotic experiences

Childhood victimisation was associated with incident PE in the complete sample (Table 3; OR = 2.64, 95% CI 1.90–3.66).

### The interaction between childhood victimisation and adult victimisation on the outcome psychotic experiences

The risk of PE in subjects with both childhood victimisation and adult victimisation was not larger than the product of their risks in participants with isolated adult victimisation and subjects with isolated childhood victimisation separately, indicating that there was no interaction between adult victimisation and childhood victimisation (Table 3).

## Discussion

### Overview of results

**Fig. 2:**
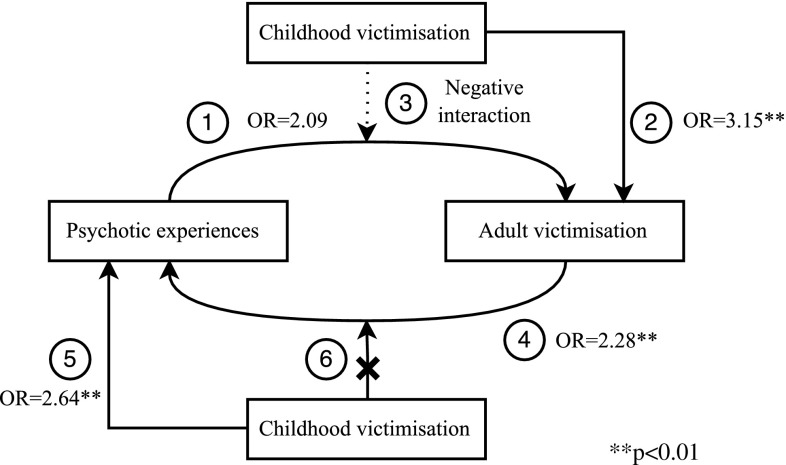
Results of testing the hypotheses relating to the bidirectional association between psychotic experiences and victimisation

To our knowledge, the present study is the first to examine the bidirectional, longitudinal associations between PE and adult victimisation using a prospective, general population sample, while also assessing the moderating effect of childhood victimisation. It was hypothesized that PE increase the risk of incident adult victimisation, and that adult victimisation increases the risk of incident PE. Moreover, it was hypothesized that childhood victimisation increases the risk of both adult victimisation and PE and that the bidirectional associations between PE and adult victimisation would be increased by the presence of childhood victimisation (Fig. 1). The present results showed evidence for the hypothesized bidirectional association between adult victimisation and PE. However, the hypothesis of a positive interaction between childhood victimisation and both PE and adult victimisation was falsified (Fig. 2).

### The association between baseline psychotic experiences and incident adult victimisation

Previous studies reported an association between psychotic disorder and adult victimisation [8, 15, 37, 39]. However, the temporal sequence of the association could not be determined in these studies because all studies used cross-sectional study designs. The present study showed that individuals with isolated PE or isolated childhood victimisation at baseline were at increased risk of any adult victimisation. However, contrary to our hypothesis that PE and childhood victimisation would act synergistically to increase the risk of adult victimisation, results showed that the co-occurrence of PE with childhood victimisation was associated with a lower risk of adult victimisation than isolated PE or isolated childhood victimisation. The results of the stratified analyses revealed that exposure to PE after childhood victimisation was not associated with adult victimisation, while PE increased the risk of adult victimisation in individuals without childhood victimisation. Similarly, exposure to childhood victimisation was not a risk factor for adult victimisation in adults exposed to PE, while childhood victimisation did increase the risk of adult victimisation in individuals not exposed to PE. Therefore, there was a negative interaction effect, pointing towards ‘parallelism’ instead of the hypothesized ‘synergism’ [40, 41], thus suggesting that PE and childhood victimisation act through competing pathways in increasing risk for adult victimisation. In other words, any excess risk for adult victimisation would already have been consumed after isolated exposure to either PE or childhood victimisation.

The results for the outcomes physical victimisation and psychological victimisation were similar to the results of the outcome of any adult victimisation, showing a trend towards a negative interaction. However, interaction could not be examined for the outcome of sexual victimisation. As opposed to the results for physical victimisation and psychological victimisation, available results show that PE were associated with incident sexual victimisation in the subgroup with childhood victimisation. However, the interaction could not be analysed. Therefore, it is possible that the association between PE and adult sexual victimisation differs from the associations between PE and other forms of adult victimisation. This finding would be in line with previous research that found that childhood sexual abuse was differentially associated with psychosis compared to other victimising experiences during childhood [9, 12, 29].

The mechanisms behind the association between PE and incident adult victimisation remain unclear. One possible explanation is that individuals with PE display disordered behaviour, for example arising from paranoid delusions, leading to social conflict and victimisation. Previous research showed that PE increase the risk of violence perpetration [36]. Therefore, it is possible that adult victimisation occurs in response to violence perpetration. Another possible explanation is that individuals with PE live in poorer social environments where adult victimisation is more likely to occur. However, the present analyses were adjusted for household income as a proxy for socio-economic status. Finally, it is possible that individuals with PE are more likely to report adult victimisation, for example because of paranoid interpretations of social interactions. To our knowledge, the reliability of adult victimisation reports in individuals with psychosis has not been studied. However, it has been shown that individuals with psychosis are able to provide reliable reports of childhood victimisation [42, 43]. Therefore, it is unlikely that the association between PE and adult victimisation can be fully attributed to differential reporting of adult victimisation by individuals with PE.

### The association between baseline adult victimisation and incident psychotic experiences

Results of the present study showed that all forms of adult victimisation were associated with the development of incident PE. This confirms our hypothesis that the association between PE and adult victimisation is bidirectional, similar to the previously reported bidirectional association between PE and childhood victimisation [10]. Consistent with previous work [10–12, 16, 44], childhood victimisation was associated with incident PE, both in the presence and absence of co-occurring adult victimisation. However, to our hypothesis of a positive interaction between childhood victimisation and adult victimisation for the outcome of incident PE, the risk of PE in subjects with both childhood victimisation and adult victimisation was not larger than the product of the isolated risks. This finding is not fully consistent with the previous literature, which showed positive interactions between childhood victimisation and adult victimisation for the outcome PE [21, 30, 32]. However, all studies used a wide variety of definitions of childhood victimisation and adult victimisation, thus impeding direct comparison. The present results show that childhood victimisation and adult victimisation are independent, cumulative risk factors for PE. This finding is relevant, since poly-victimisation is prevalent among individuals with severe mental illness [9, 15]. Moreover, this finding shows that there are two pathways from childhood victimisation to psychosis: one direct one and one indirect one through adult victimisation.

Literature on the mechanisms behind the association between adult victimisation and incident psychosis is scarce. However, both adult victimisation and childhood victimisation have been linked to various non-psychotic mental disorders [45–49]. Since PE are prevalent among individuals with non-psychotic mental disorders [18, 50] and have been identified as an indicator of severity in non-psychotic psychopathology [50–54], the link between adult victimisation and incident PE is possibly confounded by the presence of non-psychotic psychopathology [50–54]. Furthermore, it is likely that the mechanisms underlying the impact of adult victimisation on psychosis are similar to the mechanisms mediating impact of childhood victimisation on psychosis [21, 55]. Various biological and psychological processes have been associated with both psychosis and childhood victimisation [11, 56, 57]. Biological processes that link victimisation with an increase in psychosis risk include hyperactivation and sensitization of the hypothalamic–pituitary–adrenal (HPA) axis, decreased hippocampal volume, reduced brain-derived neurotrophic factor (BDNF) [56] and increased dopamine release [11]. Furthermore, victimisation may increase the risk of psychosis psychologically by contributing to the development of a worrying thinking style, negative beliefs about the self and reasoning biases such as jumping to conclusions [57]. However, more research to examine the mechanisms underlying the link between psychosis and adult victimisation is needed.

### Strengths and limitations

Strength of the present study is the prospective, longitudinal study design that enables the bidirectional assessment of psychosis and victimisation in a general population sample, while controlling for various confounders identified in the literature. Another strength is the use of clinically validated PE instead of self-reported PE [58].

The results of the study should be interpreted in the light of some limitations. First, the definitions of incident physical and psychological victimisation had to be restricted to victimisation by an intimate partner to remain consistent with the baseline definitions in the dataset. To overcome this limitation, individuals without a partner at baseline and any of the follow-up measurements were excluded from the analyses when using physical victimisation, psychological victimisation or any adult victimisation as the outcome, resulting in an exclusion of 1066 individuals for the present analysis. To our knowledge, no previous study examined whether victimisation by an intimate partner may be differently associated with psychosis than victimisation by any person. To examine this, sensitivity analyses were conducted examining the association between baseline PE and adult victimisation by any person at follow-up, while excluding individuals without a partner at baseline. The results of these sensitivity analyses were similar to the results of the main analyses in terms of effect size. However, statistical significance was greater as a result of increased statistical power. Therefore, the association between PE and adult victimisation perpetrated by any person appears to be similar to the association between PE and adult victimisation perpetrated by an intimate partner. However, more research is required to investigate this issue.

Second, baseline data on household income was missing for 604 individuals. Again, sensitivity analyses were conducted using the missing data as a separate category. Results of these analyses were similar to the main results.

Third, statistical power was low in some analyses, in particular in the interaction analyses. Thus, to screen for potential interactions, alpha for interaction effects was set at 0.10. Raising the alpha to 0.10 increases the risk of false positive results. Therefore, it is possible that some results represent type I error. More studies are required to replicate the interactions identified in this study.

Furthermore, data about baseline victimisation was collected retrospectively. Since participants in this study were aged 18–64 years, it is possible that differences in recall impacted the results. Previous studies showed evidence for age-related differential recall, with individuals underreporting victimisation as age increases [59–61]. Therefore, it is possible that the associations between victimisation and psychosis identified in this study would be stronger in the absence of age-related differential recall. In addition, the risk of exposure to adult victimisation and PE varies by age, suggesting an interaction effect between age, PE and adult victimisation. To examine this, sensitivity analyses were conducted testing for interaction effects between the various predictor variables and five age categories. Results of this analysis showed that there was no evidence for interaction between any of the predictor variables and age when studying incident PE. In addition, the association between baseline PE and incident AV did not differ between the various age groups above 25 years, but there was some evidence for a decreased association in the youngest age group (18–25 years). However, statistical power in the youngest age group was insufficient to further explore this finding. Other studies with more young adults are needed to study this hypothesis.

Finally, follow-up data was missing for 2028 participants (attrition = 30.5%). Attrition was associated with sociodemographic factors, but not with mental health status, making it unlikely that attrition would induce bias [62]. In addition, attrition was not associated with baseline PE or baseline victimisation status. Therefore, selective attrition is unlikely to have biased the results.

## Conclusions and implications

**Fig. 3:**
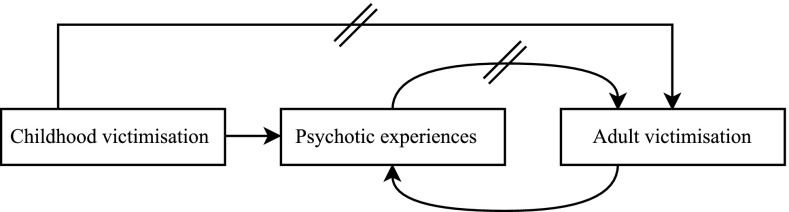
The complex interplay of psychosis and victimisation across the life course. // Points towards parallelism, i.e. childhood victimisation and psychotic experiences are competing risk factors for adult victimisation

The present study did not find evidence for all hypotheses (Fig. 2). To integrate this updated evidence, Fig. 2 was transformed into Fig. 3, removing the connections that were not supported by the present results. PE and childhood victimisation were identified as competing risk factors for adult victimisation. In addition, childhood victimisation and adult victimisation were independent, cumulative risk factors for PE. Thus, PE and adult victimisation are bidirectionally associated in individuals without childhood victimisation. In individuals with childhood victimisation, there are two pathways from childhood victimisation to PE: one direct and one indirect through adult victimisation. In conclusion, psychosis and victimisation are strongly interconnected throughout the life course, resulting in a complex interplay in which childhood victimisation and adult victimisation lead to both PE and re-victimising experiences. Because victimisation across the life course has been associated with several adverse consequences [49, 63–66], prevention strategies against victimisation, both during childhood and during adulthood, are important to prevent individuals from entering a spiral leading to mental illness and re-victimisation. Moreover, intervention programs are needed to prevent further re-victimisation in individuals who have already experienced victimisation. However, the development of adequate prevention and intervention programs requires further understanding of the mechanisms underlying the associations identified in the present study. Therefore, more longitudinal research is required to obtain a deeper understanding of the complex interplay between psychosis and victimisation across the life course.
